# Multiple Exposures to *Ascaris suum* Induce Tissue Injury and Mixed Th2/Th17 Immune Response in Mice

**DOI:** 10.1371/journal.pntd.0004382

**Published:** 2016-01-27

**Authors:** Denise Silva Nogueira, Pedro Henrique Gazzinelli-Guimarães, Fernando Sérgio Barbosa, Nathália Maria Resende, Caroline Cavalcanti Silva, Luciana Maria de Oliveira, Chiara Cássia Oliveira Amorim, Fabrício Marcus Silva Oliveira, Matheus Silvério Mattos, Lucas Rocha Kraemer, Marcelo Vidigal Caliari, Soraya Gaze, Lilian Lacerda Bueno, Remo Castro Russo, Ricardo Toshio Fujiwara

**Affiliations:** 1 Laboratory of Immunology and Parasite Genomics, Department of Parasitology, Institute of Biological Sciences, Universidade Federal de Minas Gerais, Belo Horizonte, Brazil; 2 Institute of Biological and Health Sciences, Universidade Federal do Mato Grosso, Cuiabá, Brazil; 3 Department of General Pathology, Institute of Biological Sciences, Universidade Federal de Minas Gerais, Belo Horizonte, Brazil; 4 Laboratory of Pulmonary Immunology and Mechanics, Department of Physiology and Biophysics, Institute of Biological Sciences, Universidade Federal de Minas Gerais, Belo Horizonte, Brazil; 5 Laboratory of Cellular and Molecular Immunology, René Rachou Institute, Oswaldo Cruz Foundation–FIOCRUZ, Belo Horizonte, Brazil; University of Nottingham, UNITED KINGDOM

## Abstract

*Ascaris* spp. infection affects 800 million people worldwide, and half of the world population is currently at risk of infection. Recurrent reinfection in humans is mostly due to the simplicity of the parasite life cycle, but the impact of multiple exposures to the biology of the infection and the consequences to the host’s homeostasis are poorly understood. In this context, single and multiple exposures in mice were performed in order to characterize the parasitological, histopathological, tissue functional and immunological aspects of experimental larval ascariasis. The most important findings revealed that reinfected mice presented a significant reduction of parasite burden in the lung and an increase in the cellularity in the bronchoalveolar lavage (BAL) associated with a robust granulocytic pulmonary inflammation, leading to a severe impairment of respiratory function. Moreover, the multiple exposures to *Ascaris* elicited an increased number of circulating inflammatory cells as well as production of higher levels of systemic cytokines, mainly IL-4, IL-5, IL-6, IL-10, IL-17A and TNF-α when compared to single-infected animals. Taken together, our results suggest the intense pulmonary inflammation associated with a polarized systemic Th2/Th17 immune response are crucial to control larval migration after multiple exposures to *Ascaris*.

## Author Summary

Human ascariasis caused by the helminths *Ascaris lumbricoides* and *Ascaris suum*, is the most prevalent neglected tropical disease in the world, affecting more than 800 million people and mainly school-aged children. The parasite life cycle may be divided in two distinct phases after the initial infection: (i) migration of parasitic larval stages through several tissues (intestinal mucosa, blood circulation, liver and lung/airways), namely larval ascariasis; and (ii) establishment of adult worms in the lumen of the small intestine, causing the despoilment of nutrients and secretion/excretion of parasitic products, which down modulate the immune response of the host and characterizing the chronic infection. Over the past decades special focus has been dedicated to chronic *Ascaris* infection with the assessment of immunopathological aspects of the disease in chronically infected individuals from endemic areas. However, knowledge about larval ascariasis still remains scarcedue the limitations of diagnostic techniques and the need for an experimental model that would mimic natural infection. Indeed, many aspects of the immunobiology of the early *Ascaris* infection are still poorly understood. In this context, single and multiple infections in mice were performed in order to characterize the parasitological, histopathological, tissue functional and immunological aspects of larval ascariasis.

## Introduction

New information from the Global Burden of Disease Study 2010 (GBD 2010) indicates that more than 800 million people are infected with *Ascaris* spp. (*A*. *lumbricoides* and *A*. *suum*), which ranks ascariasis as the most common affliction of people living in poverty [1]. In the past, *A*. *suum* has been implicated as an anthropozoonotic species based on epidemiological evidence from the field [2], molecular similarity to *A*. *lumbricoides* [3, 4] and also by experimental infection in humans [5]. Ascariasis is frequently associated with high rates of reinfection in endemic areas due to a constant exposure to the infective form of the parasite [6, 7].

Human ascariasis is characterized by a Th2 and regulatory immune response [8, 9], although innate production of IL-5, IL-6 and TNF-α seems to play crucial role in the pathogenesis of experimental larval ascariasis [10]. Despite the lack of evidence on *Ascaris* infection, new studies have been proposed that an IL-6-dependent, Th17 response might play an important role into the pathogenesis of helminth infections [11] and allergic manifestations [12], resulting in modulation of the Th2 response and possible susceptibility of the host to the parasitic infection. The role of IL-17 in the pathogenesis of helminth infection was highlighted in the development of hepatointestinalperioval granulomas caused by *Schistosoma mansoni* infection [13].

Larval ascariasis (established by larval migration through the host’s organs) is characterized by intense pulmonary injury and inflammatory infiltration, which is initially comprised of neutrophils during the peak of larval migration and followed by later infiltration of eosinophils and mononuclear cells [10]. The robust inflammatory response elicited by parasitic migration seems to be protective to the host [10] and might represent the establishment of concomitant immunity to new helminthic infections. Of note, epidemiological studies have demonstrated that children are more susceptible to a higher prevalence and intensity of *Ascaris* infection than adults [1, 14], implying that partial protection against the parasite is acquired over the years. However, the mechanisms underlying the susceptibility/resistance to ascariasis still remain unknown and need to be elucidated.

Therefore, the use of a murine experimental model for *Ascaris* infection is currently crucial and may provide detailed information on the biology of early *Ascaris* spp. infection. In the current study, inbred BALB/c mice were employed due to its susceptibility to *Ascaris* spp. infection [10, 15] and also to allow comparison with previous immunopathological studies [10, 15], particularly those involving subsequent challenge infection with *A*. *suum* [16, 17]. Here, we evaluated the parasitological and immunological aspects of multiple exposure to *Ascaris* infection in mice, focusing on the immunopathological mechanisms that underlie protection against larval ascariasis.

## Materials and Methods

### Experimental model

For this study, 30 BALB/c mice (male, 8 weeks old) were obtained from the Central Animal Facility from the Federal University of Minas Gerais, Brazil. Animals were subcutaneously treated (0.2% / 20 mg of live weight) with Ivermectin (Ouro Fino, Brazil), and stool examinations were regularly performed to confirm the absence of any parasitic infection.

**Fig 1:**
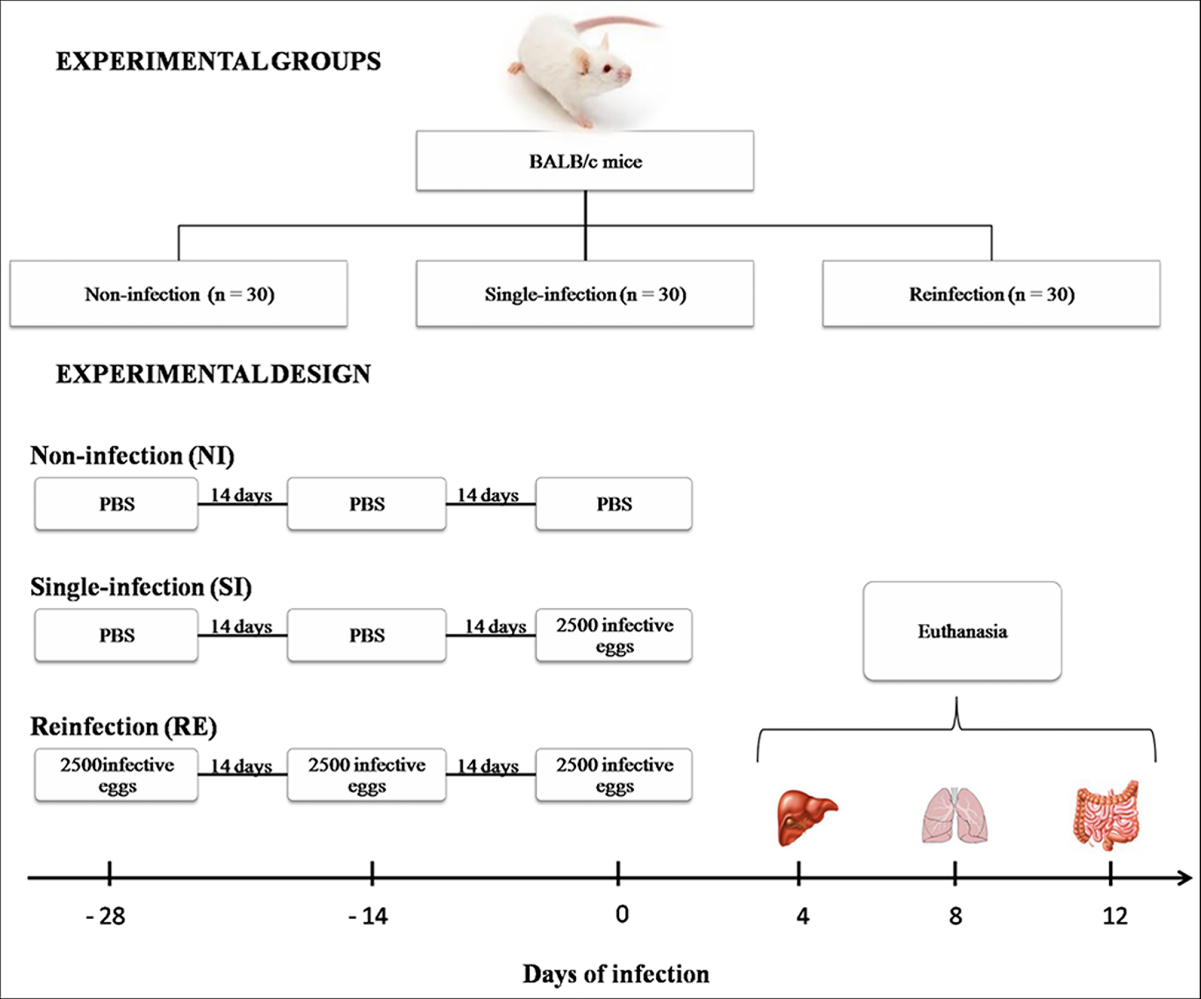
Experimental design of *Ascaris suum* single- and reinfection.

Animals were divided into three groups: the non-infection group (NI), which received PBS only; the single-infection group (SI), which received two doses of PBS and 2,500 fully embryonated *A*. *suum* eggs at the last time point; and the reinfection group (RE), where all animals received three doses of 2,500 fully embryonated *A*. *suum* eggs every two weeks (Fig 1). Tissue harvesting for parasitological and immunological evaluation was performed as previously described following the peak migration of larvae from the liver, lungs and intestine, which are observed on the 4^th^, 8^th^ and 12^th^ day of infection, respectively [10].

### Parasites

Adult *A*. *suum* worms were harvested from pigs at a Brazilian slaughterhouse (Belo Horizonte, Minas Gerais, Brazil). Eggs were isolated from uteri of female worms by gentle mechanical maceration and further purified by use of cell strainers (70 μm). Isolated eggs were incubated with 0.2 M H_2_SO_4_ for embryonation, as described by Boes and colleagues [18]. After the 100^th^ day of culture, which corresponds to the peak of larval infectivity [10], fully embryonated eggs were used in experimental infections.

### Experimental infection and parasitological analysis

Using a gavage needle, all animals received either 200 μl of PBS or 2,500 embryonated eggs in 200 μl PBS, according to the group or timepoint. Parasite burden was evaluated by recovery of larvae from liver (n = 6/group), the lungs (n = 6/group), and the small intestine (n = 6/group). Tissues were collected, sliced with scissors and placed in a Baermann apparatus for 4 hours in the presence of PBS at 37°C. The recovered larvae were then fixed (1% formaldehyde in PBS) and counted under an optical microscope.

### Haematological analysis

The haematological profiles of infected and reinfected mice were evaluated during the different stages of the larvae migration. Briefly, 500 μL of blood was collected from a superficial vein of 6 BALB/c mice per group using capillary Pasteur pipettes primed with anticoagulant EDTA. The total leukocytes were counted using an automated hematological analyzer (Bio-2900 Vet, Bioeasy, USA) and percentages and absolute numbers of lymphocytes, monocytes, eosinophils and neutrophils were further determined by optical microscopy in blood smears stained with Giemsa.

### Cytokine profile

In order to determine the cytokine profile in the serum, 500 μl of blood was collected from each mouse at all experimental timepoints. Blood was collected from the retro-orbital sinus using a capillary Pasteur pipette without anticoagulant. Collected blood was transferred to Eppendorf tubes for coagulation, followed by centrifugation and serum collection. The production of IL-2, IL-4, IL-6, IL-10, IL-17A, IFN-γ and TNF-α was assessed by flow cytometry (Th1/Th2/Th17 Cytometric Bead Array, BD Biosciences, USA) using a FACScan (BD Biosciences, USA) according to the manufacturer's recommendation. Serum levels of IL-5 were measured using a sandwich ELISA kit (R&D Systems, USA) according to the manufacturer's instructions. The absorbance was determined by a VersaMax ELISA microplate reader (Molecular Devices, USA) at a wavelength of 492 nm, and the cytokine concentration (pg/mL) for each sample was calculated by interpolation from a standard curve.

### Eosinophil peroxidase and neutrophil myeloperoxidase assays

The activities of eosinophil peroxidase (EPO) and neutrophil myeloperoxidase (MPO) in the lung homogenates were measured according to a method described by Strath and modified by Silveira [19, 20]. After tissue homogenization (Power Gen 125 –Fisher Scientific Pennsylvania, USA), the homogenate was centrifuged at 8,000 g for 10 min at 4°C and the remaining pellet was examined to determine the activity of EPO and MPO. For the EPO assay, the pellet was homogenized in 950 μL PBS and 0.5% hexadecyltrimethylammonium bromide (Sigma Chemical Co, St. Louis, MO, USA) and then frozen/thawed three times using liquid nitrogen. The lysate was then centrifuged (1,500 g, 4°C, 10 min) and the supernatant was distributed (75 μL/well) in a 96-well microplate (Corning, USA) followed by the addition of 75 μL of substrate (1.5 mM OPD and 6.6 mM H_2_O_2_ in 0.05 M Tris-HCl, pH 8.0). After incubation for 30 minutes at room temperature, the reaction was stopped by the addition of 50 μL of 1 M H_2_SO_4_ and the absorbance was determined at 492 nm.

For the MPO assay, the pellet was homogenized in 200 μL of buffer 1 solution (0.1 M NaCl, 0.02 M Na_3_PO_4_, 0.015 M Na_2_EDTA, pH 4.7) followed by centrifugation (1,500 g, 4°C, 10 min). 800 μL of buffer 2 solution (0.05 M NaPO_4_, 0.5% hexadecyltrimethylammonium bromide) was added to the pellet and the mixture was homogenized and then frozen/thawed three times using liquid nitrogen. The lysate was centrifuged (1,500 g, 4°C, 10 min), and the supernatant was used for the enzymatic assay. 25 μL/well were distributed on to 96-well microplates (Corning, USA) followed by the addition of 25 μL of substrate TMB (3.3'-5.5;- tetramethylbenzine + 1.6 mM dimethylsulfoxide) and 100 μL of 0.5 M H_2_O_2_. After incubation for five minutes at room temperature, the reaction was stopped by the addition of 100 μL of sulphuric acid (1 M H_2_SO_4_). Absorbance was determined by a VersaMax ELISA microplate reader (Molecular Devices, USA) at a wavelength of 450 nm.

### Bronchoalveolar lavage

For the analysis of the cellularity and blood loss in the bronchoalveolar lavage (BAL), six single- and reinfected BALB/c mice were euthanized eight days after infection. Basically, a 1.7 mm catheter was inserted into the trachea of the animals, and 1 mL of PBS was used twice for perfusion and aspiration in order to assess the leukocyte infiltration in the bronchoalveolar compartment. The bronchoalveolar lavage was filtered on cell strainers 70 μm (BD, USA) to recover and quantify the *A*. *suum* larvae presented in the BAL. The material was centrifuged at 3,000 g for 10 minutes and the pellet was used to determine the total number of leukocytes and differentiation of macrophages, lymphocytes, eosinophils and neutrophils using optical microscopy. The supernatant was used to quantify the amount of total protein and haemoglobin content. Samples from six non-infected BALB/c mice were used as controls.

The quantification of total protein was determined by BCA Protein Assay kit (Thermo Scientific, USA) and was performed on BAL to measure possible protein leakage into the airways, as previously described [21]. The results were expressed as μg of total protein per mL of BAL. The extent the alveolar haemorrhage was assessed by the amount of hemoglobin (Hb) detected in BAL supernatant using the Drabkin method, as previously described [22]. The hemoglobin concentration in the samples was determined spectrophotometrically by measuring absorbance at 540 nm and interpolation from a standard hemoglobin curve, starting at 1 mg/mL. Hemoglobin content was expressed as μg of Hb per mL of BAL.

### Histopathological analysis

For the liver and lung histological analysis, organs were removed on the fourth and eighth days after infection and were fixed in a 10% solution of formaldehyde (Synth, Brazil) in PBS for 72 hours. After processing in alcohol and xylol, tissue fragments were embedded in paraffin, and 4 μm thick sections were obtained and stained with hematoxylin and eosin (H&E). The tissues were analyzed using KS300 software coupled to a Carl Zeiss image analyzer (Oberkochen, Germany).

Severity of liver injury was assessed by calculation of all areas of inflammation and necrosis by morphometric analysis. Hepatic lesions were assessed through a 10x objective microscope Axiolab Carl Zeiss and the images were captured using a JVC TK-1270/RGB microcamera (Tokyo, Japan). The area of lesions was measured in μm^2^ using KS300 software coupled to Carl Zeiss image analyzer (Oberkochen, Germany). All slides were digitized by scanner Canon Lide 110 at 300 dpi resolution. The pixels of each histological section were fully screened, with subsequent creation of a binary image and calculating the total area of the cut. The area of the lower cutoff was used as a minimum standard of tissue to be statistically analyzed [23].

To evaluate the intensity of pulmonary inflammation and hemorrhage, the degree of thickening of interalveolar septa was calculated. Thirty random images were captured at 40x objective, comprising an area of 1,6x10^6^ mm^2^. Through the KS300 software, all pixels of the lung tissue in the real image were selected to create a binary image, digital processing and calculating the area in mm^2^ of the interalveolar septum [24].

### Assessment of respiratory mechanics

Mice were anesthetized with a subcutaneous injection of ketamine and xylazine (8.5 mg/kg xylazine and 130 mg/kg ketamine) to maintain spontaneous breathing under anesthesia. Mice were tracheostomized, placed in a body plethysmograph and connected to a computer-controlled ventilator (Forced Pulmonary Maneuver System, Buxco Research Systems, Wilmington, North Carolina USA). This laboratory set-up, specifically designed for use on mice, has only a canula volume (death space) of 0.8 mL and provides semi-automatically three different maneuvers: Boyle’s Law FRC, quasi-static pressure-volume and fast-flow volume maneuver. First, an average breathing frequency of 160 breaths/min was imposed to the anesthetized animal by pressure-controlled ventilation until a regular breathing pattern and complete expiration at each breathing cycle was obtained. Under mechanical respiration the Dynamic Compliance (Cdyn) and Lung Resistance (Rl) were determined by Resistance and Compliance RC test. To measure the Forced Vital Capacity (FVC) and Inspiratory Capacity (IC), the quasi-static pressure-volume maneuver was performed, which inflates the lungs to a standard pressure of +30 cm H_2_O and then slowly exhales until a negative pressure of -30 cm H_2_O is reached. The quasi-static chord compliance (from 0- to +10 cm H_2_O) was calculated with this maneuver considering the volume/pressure of the expiration. With the fast flow volume maneuver, lungs were first inflated to +30 cm H_2_O and immediately connected to a highly negative pressure in order to enforce expiration until -30 cm H_2_O. Forced Expiratory Volume (forced expiratory volume at 100 milliseconds, FEV100) was recorded during this maneuver. Suboptimal maneuvers were rejected and for each test in every single mouse at least three acceptable maneuvers were conducted to obtain a reliable mean for all numeric parameters.

### Statistical analysis

**Fig 2:**
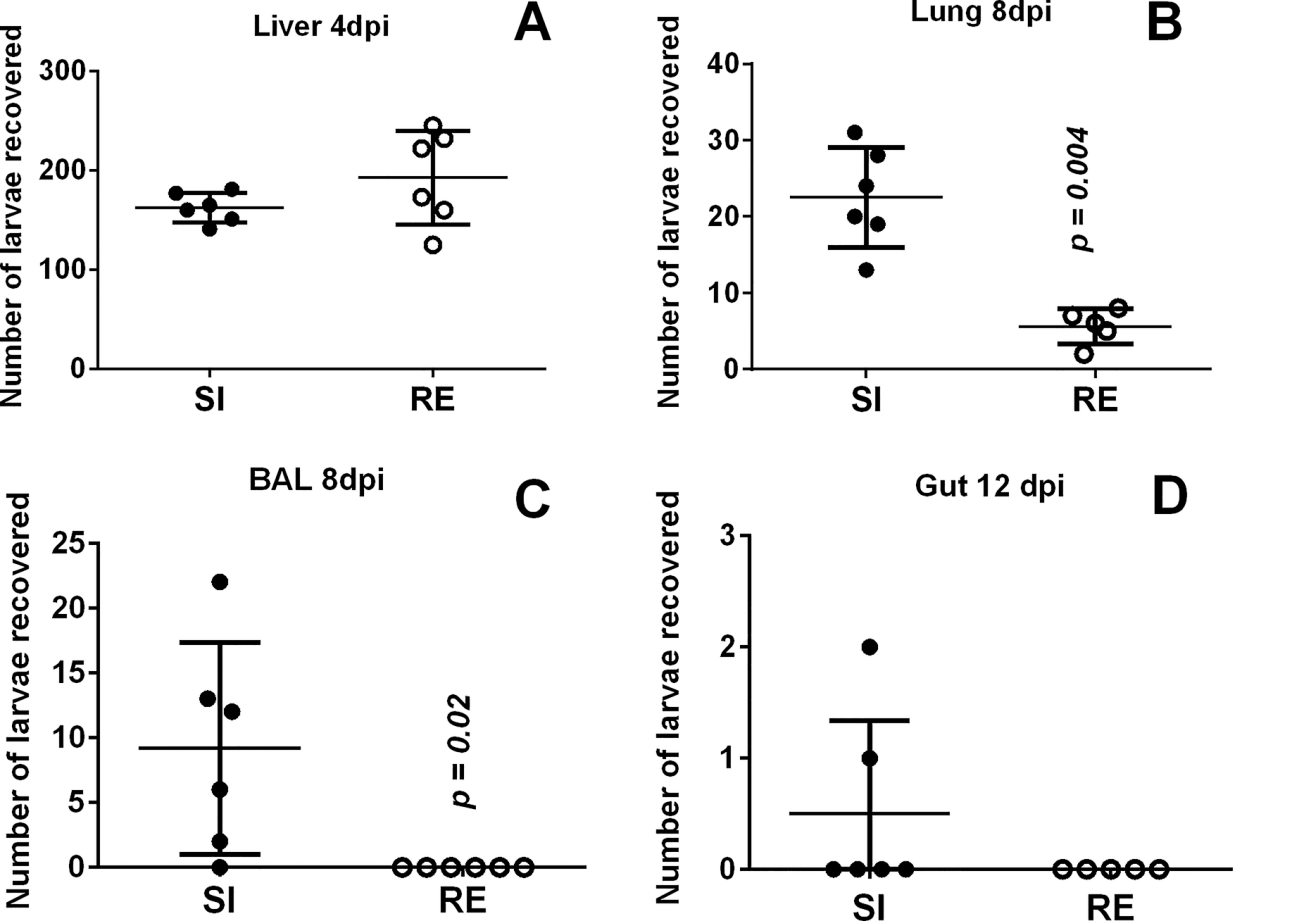
Number of larvae recovered from host organs. (A) Liver on the 4^th^ day post-infection; (B) lung on the 8^th^ day post-infection; (C) BAL on the 8^th^ day post-infection; and (D) gut on the 12^th^ day post-infection. Filled circles–single infection (SI) group; Open circles–reinfection (RE) group. Mann-Whitney test was used to assess differences between groups and are depicted in the graphs by the p values.

**Fig 3:**
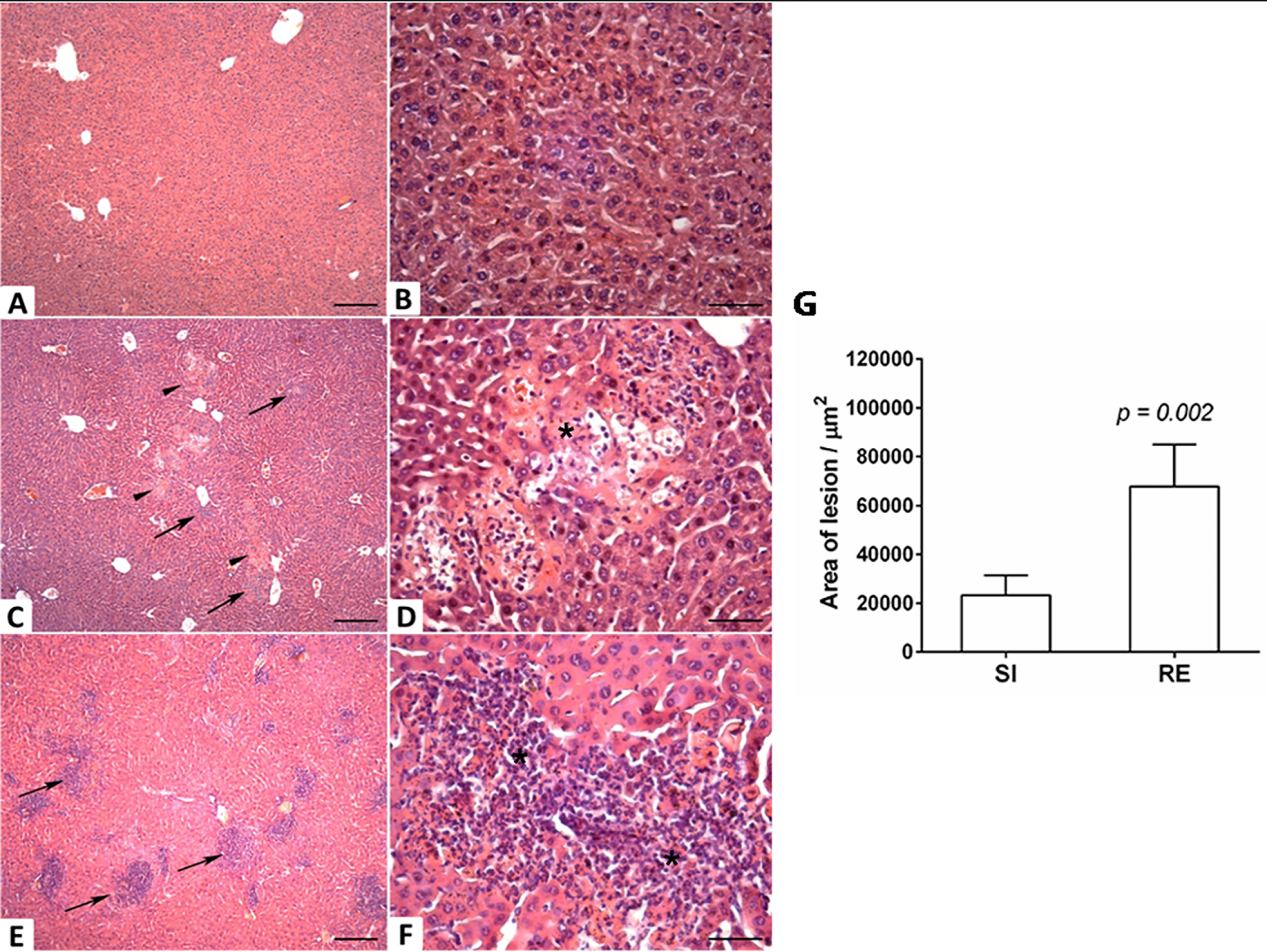
Histopathological visualization of lesions caused by larval migration in the liver on the 4^th^ day post-infection and area of the lesions caused by larval migration. (A and B) Non-infected mouse; (C and D) Single-infected mouse with the presence of necrosis (arrowheads) and mild inflammatory infiltrate (arrows); (E and F) Reinfected mouse with presence of intense inflammatory infiltrate (arrows) and necrosis (*). Lower magnification Bar scale = 50 μm. Higher magnification Bar scale = 200 μm. (G) Area of lesion caused by larval migration in liver on the 4^th^ day post-infection. Mann-Whitney test was used to evaluate differences between groups.

**Fig 4:**
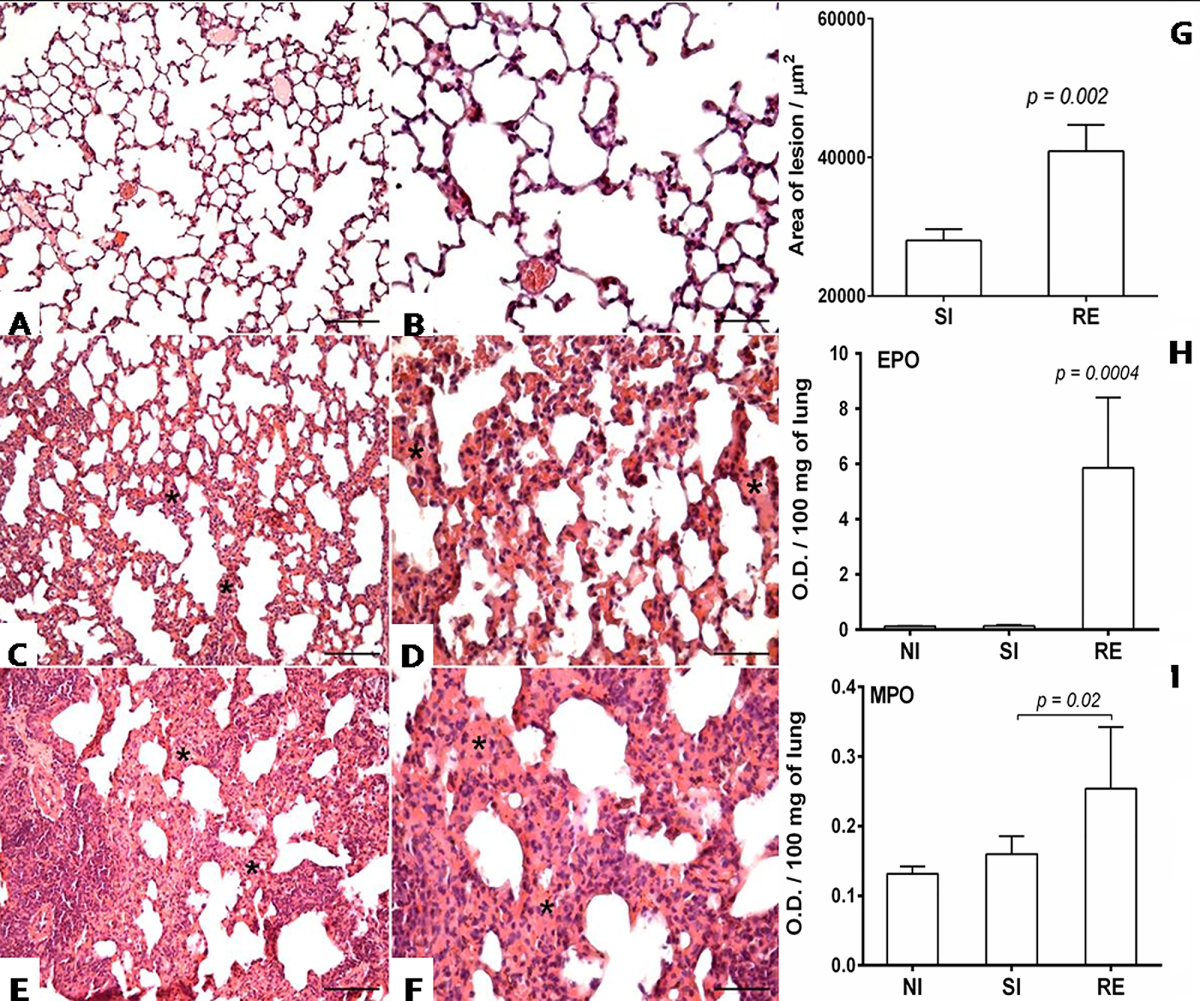
Histopathological visualization of the lesion caused by larval migration in the lungs on the 8^th^ day post-infection and area of the lesion caused by larval migration. (A and B) Non-infected mouse; (C and D) Single-infected mouse with slight thickening of the septum at the expense of inflammatory infiltrates, hyperaemia and haemorrhage (*); (E and F) Reinfected mouse with inflammatory infiltrate, intense hyperaemia and haemorrhage, causing extensive thickening of the septum (*). Lower magnification Bar scale = 50 μm. Higher magnification Bar scale = 100 μm. (G) Area of the lesion caused by larval migration and inflammation in the lung on the 8^th^ day post-infection; Mann-Whitney test was used to assess differences between the groups. (H-I) Optical density representing the MPO and EPO activity in the lung at 8 days post-infection. (H) EPO production in the lung on the 8^th^ day post-infection. (I) MPO production in the lung on the 8^th^ day post-infection. Kruskal-Wallis test followed by Dunn´s multiple comparisons test was used to evaluate differences between groups. The p values in the graphs represent the significant differences.

**Fig 5:**
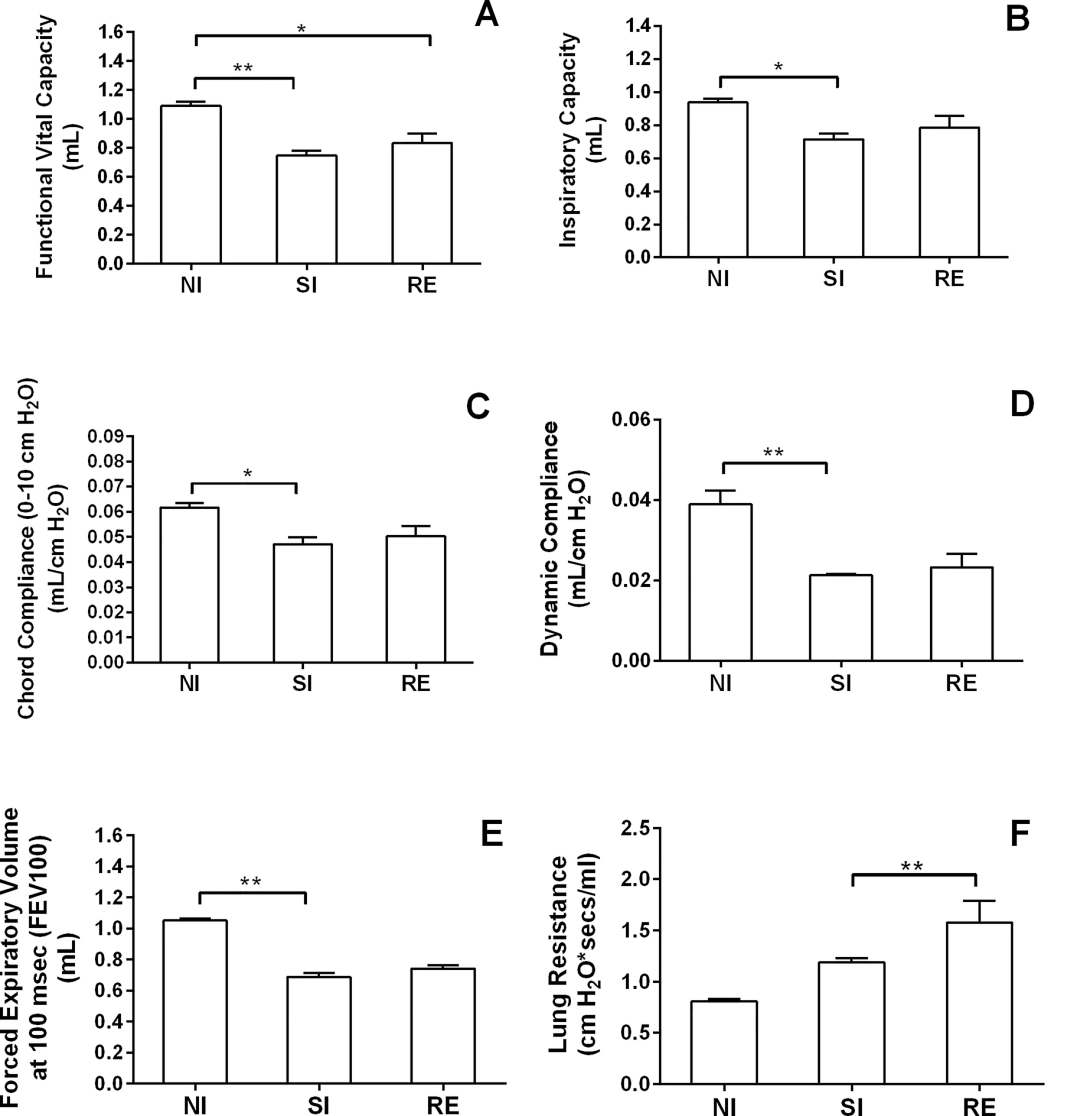
Assessment of lung mechanics after single or multiple *Ascaris* infection in mice. Forced spirometry was performed to investigate the injury by modifications in lung functions. The parameters assessed were Functional Vital Capacity (A), Inspiratory Capacity (B), Dynamic Compliance Forced (C), Chord Compliance (D), Expiratory Volume at 100 msec (E) and Lung Resistance (F). Kruskal-Wallis test followed by Dunn´s multiple comparisons test was used to evaluate differences among groups. Results are shown as the mean ± SEM. * *p* < 0.05; ** *p* < 0.01.

**Fig 6:**
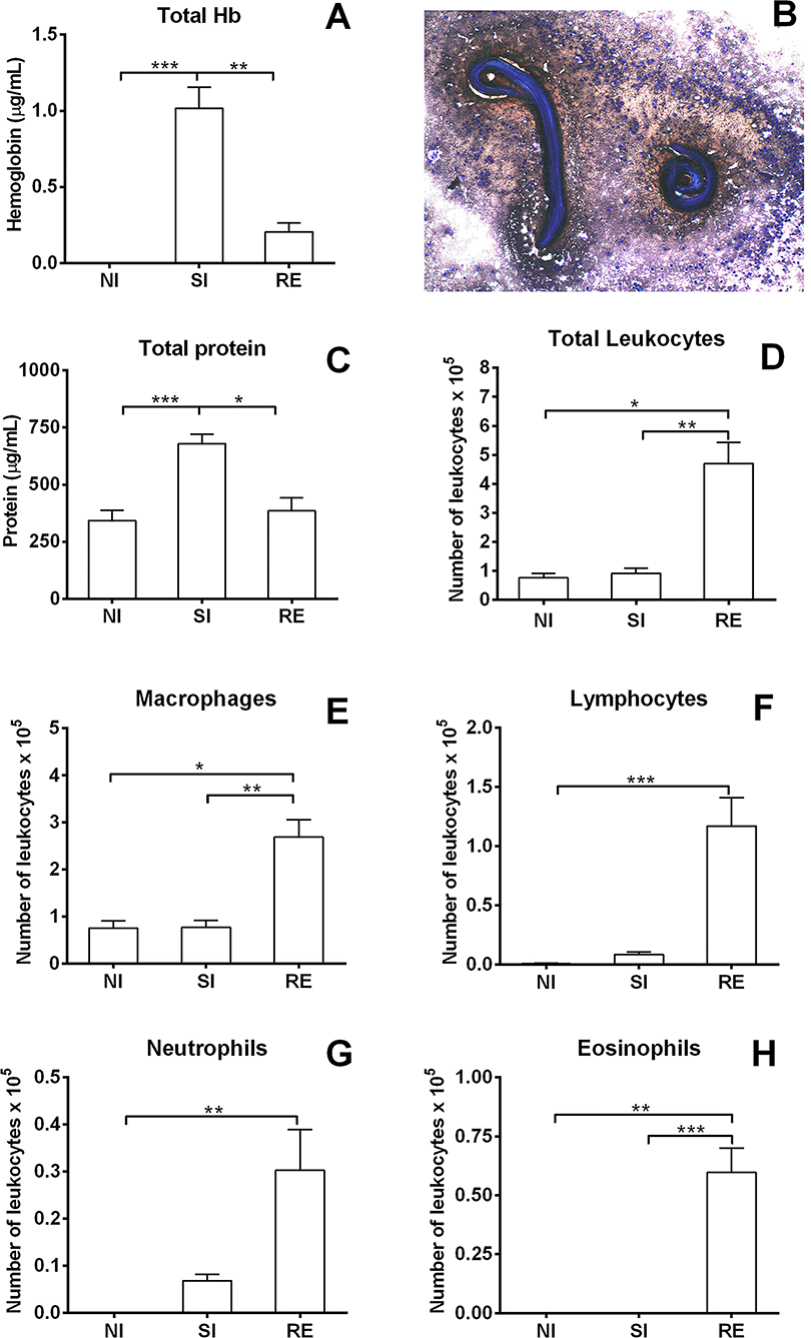
Levels of haemoglobin, total protein, mononuclear and granulocyte cell counts in the BAL on the 8^th^ day post-infection. (A) Haemoglobin levels in BAL on the 8^th^ day post-infection. (B) Larvae from *A*. *suum* surrounded by leukocytes in the BAL 8 days post-infection. (C) Total protein levels in the BAL on the 8^th^ day post-infection. (D) Total leukocytes counts in the BAL on the 8^th^ day post-infection. (E) Macrophage counts in the BAL. (F) Lymphocyte cell counts in the BAL. (G) Neutrophil cell counts in the BAL. (H) Eosinophil cell counts in the BAL. Kruskal-Wallis test followed by Dunn´s multiple comparisons test was used to evaluate differences among the groups. Results are shown as the mean ± SEM and were represented * and # for was used where * *p*< 0.05; ** *p*< 0.01 and *** *p*< 0.001 for the differences among all groups in the respective time; and # *p*< 0.05 and ## *p*<0.01 for differences to the control group at the same time.

**Fig 7:**
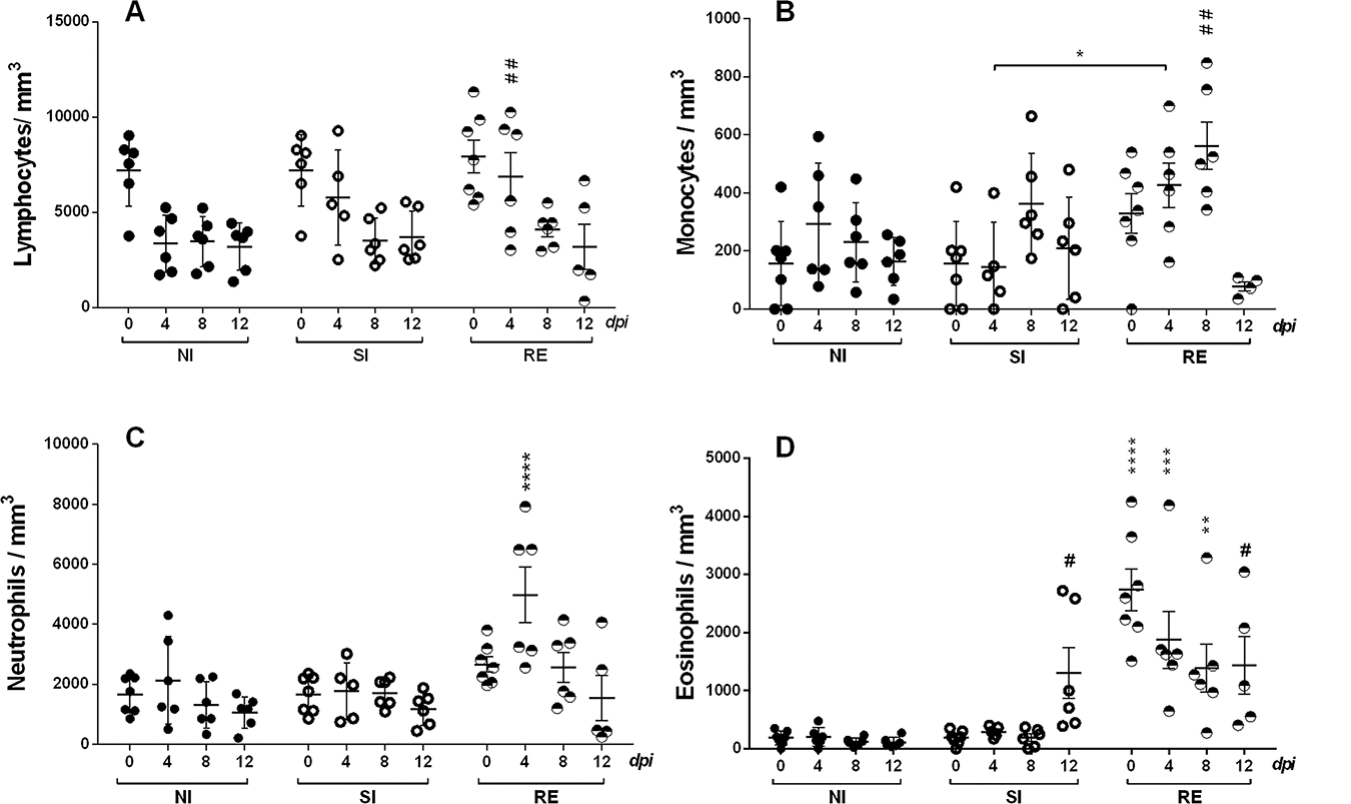
Mononuclear and granulocyte cell counting in the blood at different time points after *A*. *suum* experimental infection. (A) Lymphocyte cell counts. (B) Monocyte cell count. (C) Neutrophil cell counts. (D) Eosinophil cell counts. Filled circles–non-infected group; Open circles–single-infected group; and divided circles–reinfected group. Two-way ANOVA test followed by multiple comparison test were used to compare the variances between the groups. Results are shown as the mean ± SEM and were represented ‘*’ and ‘#’. * *p*< 0.05; ** *p*< 0.01 *** *p*< 0.001 and **** *p*< 0.0001 represent the differences between all groups in the respective time; and # *p*<0.05 and ## *p*<0.01 represent the differences to the non-infected group.

**Fig 8:**
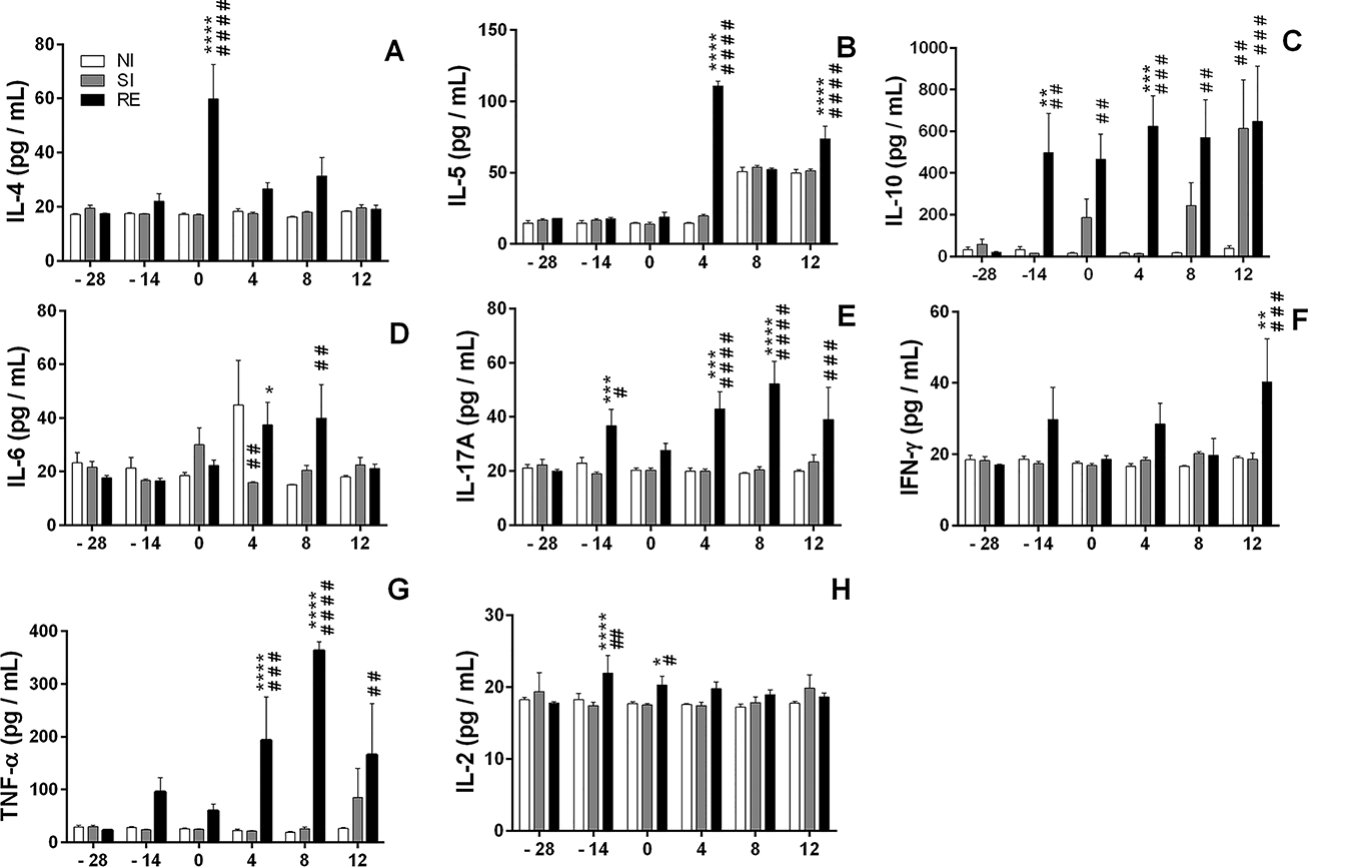
Systemic cytokine production profile from the first day to 12 days post-infection. (A) IL-4, (B) IL-5, (C) IL-10, (D) IL-6, (E) IL-17A, (F) IFN-γ, (G) TNF-α and IL-2 (H). Two-way ANOVA test followed by multiple comparisons test were used to compare the variances between the groups. Results are shown as the mean ± SEM and were represented by ‘*’ and ‘#’. * p< 0.05, ** p< 0.01, *** p< 0.001; and **** p< 0.0001 are indicated to represent the differences compared with single-infected group in the respective time; and # p< 0.05, ## p< 0.01, ### p <0.001, and #### p< 0.0001 represent the differences compared with non-infected group.

Statistical analyses were performed using the software GraphPad Prism 6 (GraphPad Inc., USA). Grubb's test was used to detect possible outliers in the samples. For comparison of parasitic burden (Fig 2) and areas of lesion in the liver and lungs (Figs 3G and 4G), the Mann-Whitney test was used. Data from EPO (Fig 4H) and MPO (Fig 4I) assays and also from pulmonary mechanics (Fig 5), haemoglobin, protein levels and BAL cellularity (Fig 6) were analysed by Kruskal-Wallis test followed by Dunn's test. Finally, Two-way ANOVA with multiple comparisons test was performed to assess differences between the groups in function of time (Figs 7 and 8). All tests were considered significant when the p value was equal or less than 0.05.

### Ethics statement

The maintenance and use of animals were in strict accordance with the recommendations of the guidelines of the Brazilian College of Animal Experimentation (COBEA). The protocol was approved by the Ethics Committee for Animal Experimentation (CETEA) of the Universidade Federal de Minas Gerais, Brazil (Protocol# 45/2012). All efforts were made to minimize animal suffering.

## Results

### Reinfection with *A*. *suum* is associated with a reduction in parasitic burden during the lung-stage larval migration

Based on a previous study from our group, we sought to determine whether multiple exposures to *A*. *suum* influenced the number of larvae recovered from liver, lung and small intestine. While no differences in the number of larvae recovered in the liver from single or multiple infection was observed (Fig 2A), the reinfected group showed a significant reduction in the number of larvae recovered from the lungs (p = 0.004) and BAL (p = 0.02) when compared to single-infected group at the 8^th^ day post-infection (Fig 2B and 2C). Following the life cycle, no significant differences in the larvae recovered in the intestine from both infected and reinfected groups were observed at 12 days post-infection (Fig 2D).

### Reinfection with *A*. *suum* induces an intense tissue inflammation that might be associated with larval control in the lungs, but consistently impairs pulmonary function

After we confirmed that multiple exposures to *A*. *suum* induced significant protection indicated by a reduction of parasite burden in the lungs, we further evaluated the pattern of cellular response by histopathological analysis in order to try to explain the mechanisms of this protection.

Although there were no differences in the numbers of larvae recovered from the liver on the 4^th^ day post-infection between single- and multiple-infected animals, the latter group showed a larger lesion area caused by larvae migration when compared to the single-infected group (p = 0.002) (Fig 3G). In the microscopical analysis of liver parenchyma, areas with hepatocyte necrosis and polymorphonuclear inflammatory infiltrate–composed primarily of eosinophils and neutrophils–were observed in the single-infected group (Fig 3C and 3D). These findings were even more pronounced in the reinfection group, in which granulomas were also present (Fig 3E and 3F).

The lungs of animals from both single- and reinfection groups showed microscopic lesions in the lung parenchyma that were characterized by the presence of a polymorphonuclear inflammatory infiltrate consisting primarily consisted of eosinophils and also thickening of the interalveolar septa when compared to controls (Fig 4A–4F). Despite the significant reduction of parasite burden in the reinfected group, the area of pulmonary lesion was considerably higher in this group when compared to the single-infected animals (p = 0.002) (Fig 4G).

Of note, the thickening of the septa was less pronounced in the single-infection group (Fig 4C and 4D) compared with the reinfection group (Fig 4E and 4F), suggesting that multiple parasitic exposures lead to chronic lung injury associated with tissue remodeling. The chronic activation of eosinophils and neutrophils in lung tissue was evident in the results of assessment of eosinophil peroxidase and myeloperoxidase activities at 8 days post-infection in the lung, which was significantly higher during reinfection when compared to single exposure to the parasite (p = 0.0004 and p = 0.02, respectively) (Fig 4H and 4I). Collectively, these data indicate that multiple exposures to *Ascaris* spp. induced a chronic and robust immune response in the lungs of reinfected group, ultimately related to increased tissue damage and protection against progression of the parasitic cycle.

The analysis of pulmonary mechanics during inflammation was performed by forced spirometry technique to further investigate the physiological modifications caused in lung functions after 8 days of single or multiple *Ascaris* infection (Fig 5). The pulmonary test detects different types of physiologic parameters in mouse lungs: (i) lung volumes to determine the effects of tissue damage by evaluation of lung volume loss, mostly caused by aedema and airway thickness, as presented by Functional Vital Capacity (Fig 5A) and Inspiratory Capacity (Fig 5B); (ii) evaluation of elastic properties of lung tissue is a measure of the lung's ability to stretch and expand by measuring the compliance (Compliance = ΔVolume/ΔPressure), which is assessed as Static lung compliance (Cchord) (change in volume for any given applied to pressure point of curve from 0 to +10 Cm H_2_O) (Fig 5C) and Dynamic lung compliance (Cdyn), which is the compliance of the lung at any given time during actual movement of air (Fig 5D); (iii) Forced Expiratory Volume at 100 msec (FEV100), which is the volume exhaled during the first 100 milliseconds of a forced expiratory maneuver started from the level of Total Lung Capacity (it is the standard index for assessing and quantifying airflow movement into the lungs); and (iv) Lung resistance, which is the resistance of the respiratory tract to the airflow movement during normal inspiration and expiration, where Rl = [(Atmospheric Pressure–Alveolar Pressure)/V] (Fig 5F).

By using Forced Pulmonary Maneuvers, we observed that single or multiple *Ascaris* infections in mice caused loss of respiratory area induced by aedema and lung septa thickening, as indicated by significant reduction in Functional Vital Capacity (Fig 5A) and Inspiratory Capacity (Fig 5B) values when compared to respective controls. Concerning the assessment of pulmonary elasticity by lung compliance and resistance analysis, we detected that infected animals displayed reduced Chord Compliance (Fig 5C) and Dynamic Compliance (Fig 5D) indicating modification in pulmonary extracellular matrix components. Moreover, infected mice presented alterations in respiratory airway flow 8 days after single or multiple infections with decreased Forced Expiratory Volume at 100 milliseconds (FEV100) when compared to controls (Fig 5E), suggesting that lung injury induced by *Ascaris* reduced the airflow into airways. A progressive elevation in Lung Resistance according to the number of infections (single or three infections) was detected (Fig 5F), indicating loss of pulmonary elasticity in both groups, but more pronounced in reinfected mice. Together, our data shows that infected animals showed alteration in airway flow (Fig 5C), loss of respiratory area (Fig 5A and 5B) and reduction of tissue elasticity (Fig 5D–5F) induced by worm migration into airways and subsequent tissue damage. Ultimately, our data suggests that altered lung functions may occur by induction of chronic lung injury and immune responses against *Ascaris* spp.

The analyses of leukocytes in the BAL of single- and reinfected mice on the 8^th^ day post-infection indicated airway haemorrhage compared with the non-infected animals (Fig 6). The presence of bleeding was higher in the single exposured animals than in reinfected group, which is consistent with the significantly higher haemoglobin levels observed in the BAL of single-infection group animals (Fig 6A), also associated with worm influx into airways (Figs 2C and 6B). Moreover, the levels of total protein in BAL were higher in the single-infection group (Fig 6C), which might also be related to increased worm transmigration and haemorrhage in BAL. In contrast, BAL of reinfected animals presented a substantial increase in the number of total leukocytes (Fig 6D).

The evaluation of differential cell counting in the BAL reinforced the notion that reinfection induces a chronic lung inflammation as observed by increased number of granulocytes in airways, and also significant augmentation of cells from adaptive immunity as phagocytes and lymphocytes, as depicted by cytospin preparations, when compared to the remaining groups (Fig 6E–6H). Our data suggest that single-infection induces acute tissue damage, followed by haemorrhage and exudation related to increased worm transmigration into airways, while reinfection might elicit a pulmonary immune response against *Ascaris* spp. resulting in a decreased number of worms present in the airways.

### Reinfection with *A*. *suum* elicits an increased number of circulating inflammatory cells and production of systemic Th2/Th17 cytokines during larval ascariasis

To elucidate the immunopathological mechanisms involved in protection against reinfection, peripheral blood from both single- and reinfected animals was collected on days zero, 4, 8 and 12 post-infection and the systemic immune responses were evaluated. Differences in the time of infection (0, 4, 8 and 12 days post-infection), the type of infection (single or multiple infection) and the interaction between these two factors were evaluated in all groups. Both factors (time and type of infection) contributed to the observed differences in the count of circulating monocyte and eosinophils, which were significantly augmented in the reinfected groups according to the progression of infection until the 8^th^ day of infection (where the peak of the parasitism in the lungs is reached).

As observed in the BAL (Fig 6), an increased number of leukocytes were detected in the blood (Fig 7). Remarkably, significant differences were observed in the number of circulating lymphocytes on the 4^th^ day of reinfection (Fig 7A) and monocytes on the 4^th^ and 8^th^ days of reinfection (Fig 7B), which were higher in the reinfected animals when compared to the single infection and control animals. While the number of circulating neutrophils was significantly higher in the reinfected animals on the 4^th^ day of infection (Fig 7C), the eosinophil counts were increased in the 12^th^ day post-infection in the single infected group and on all evaluated days to the animals that received three experimental infections (Fig 7D).

Concerning the systemic cytokine profile during reinfection (Fig 8), production of Th2 cytokines were detected only after a third exposure to the parasite, with significant production of IL-4 on day 0 and IL-5 on days 4^th^ and 8^th^ of the study (Fig 8A and 8B, respectively). Higher levels of IL-10 were detected on day -14 (after second exposure to *A*. *suum* infection) and were sustained until the end of the study (Fig 8C). Production of inflammatory cytokines was also observed, with detection of significantly higher production of IL-6 from the 4^th^ day after the third infection (Fig 8D). Of note, the most striking finding demonstrated that reinfected mice presented higher levels of IL-17A when compared with single-infected and control animals (Fig 8E).

Finally, while production of IFN-γ was significantly higher in the reinfected group only at the late stage of the third infection (12^th^ days) when compared to single-infected and control animals (Fig 8F), TNF-α production followed the IL-17A profile, where reinfected animals presented higher cytokine production from the 4^th^ day after the multiple exposure to the parasite (Fig 8G). IL-2 production was also higher in the reinfected group compared to the single- and non-infected group at the -14 and 0 day of time points (Fig 8H).

## Discussion

Recently, Gazzinelli-Guimarães et al. [10] using the BALB/c mouse strain have characterized the full pattern of *A*. *suum* larval migration and highlighted the immunopathological changes in lung tissue triggered by the larvae during a primary larval ascariasis. In the present work, the parasitological and immunological aspects of *Ascaris* spp. infection in mice were evaluated, comparing single and multiple infections and focusing on the possible mechanisms that control the protection against larval ascariasis. Indeed, several animal studies have shown that prior exposure to helminths can induce protection against reinfection to *A*. *suum* [25], *Strongyloides ratti* [26], *Neodiplostomum seoulensis* [27, 28], *Clonorchis sinensis* [29] and *S*. *stercoralis* [30], amongst other helminths. The current study also provides strong evidence that multiple exposures to *A*. *suum* induces partial protection during larval migration through lung tissue and particularly corroborate previous findings in calves where a decrease in the number of larvae was observed after a second exposure to the parasite [25].

Larval ascariasis starts precisely after *Ascaris* sp. larvale hatch in the host’s small intestine and it is characterized by progressive larval migration through the large intestine mucosa, blood circulation, liver, lungs and airways and finally back to the small intestine where they mature as adult worms, leading to a chronic and a long-term infection. Recently, an important anti-helminthic role played by eosinophils in intestine mucosal defense against invading *A*. *suum* larvae was demonstrated [31]. However, the mechanisms of protection developed by the host during hepato-tracheal migration of *A*. *suum* larvae were not fully elucidated. In this study, it was shown that pulmonary immune responses represent a crucial role to prevent reinfection, and the number of migrating larvae was reduced consistently only when the larvae reach the lungs and airways.

Associated with the control of larval migration, the multiple exposures to *A*. *suum* elicited an increased cellularity in the BAL determined by adaptive immune cells (lymphocytes and macrophages) and an intense eosinophilic and neutrophilic pulmonary inflammation that might ultimately lead to a severe impairment of the respiratory function. Moreover, the multiple exposures to *Ascaris*, apart from the expansion in the number of circulating inflammatory cells (mainly eosinophils and neutrophils), also induced a polarized Th2/Th17 lymphocyte response defined by higher levels of systemic cytokine production of IL-4, IL-5, IL-10, IL-6, TNF-α and IL-17A in comparison to single-infected animals.

Animals with multiple exposures to the parasite exhibited peripheral eosinophilia at all of the evaluated (0, 4, 8 and 12) time points, a significant increase of eosinophils in bronchoalveolar lavage fluid and higher EPO activity in the lung on the eighth day after the last infection. Moreover, the inflammatory infiltrate in the lungs was composed primarily of eosinophils and they were more prominent in the reinfected group. While an increased activity of eosinophils in mice reinfected with *S*. *stercoralis* was not directly associated with destruction of parasitic larvae [30], in our study we observed that a remarkable rise in count and activity of eosinophils was followed by a substantial reduction in the parasitic burden, suggesting the importance of eosinophils in the clearance of *Ascaris* infection [32]. On the other hand, the presence of eosinophils would be implicated in tissue repair and remodeling due to the extensive lung injury and hemorrhage associated with migration of larvae into airways, which lead to alveolar edema and robust inflammation and to further changes in pulmonary functions detected by spirometry. Of note, the reinfection induced larger areas of lung when compared with animals from the single-infection group, suggesting also that multiple exposures could lead to repeated tissue injury and chronic inflammation, causing progressive loss of respiratory function. The paradoxical patterns of immune response in lungs of reinfected animals, where significant inflammatory responses and decrease of parasitic burden are observed, suggest the possible beneficial effect of inflammation for helminth protection during pulmonary migration. The controversial contribution of the inflammatory response to the control of parasitic burden has been demonstrated in animal models of susceptibility or resistance to a single exposure to the parasite [33, 34]. Severe pulmonary inflammation was considered not important to the control of *A*. *suum* infection but directly associated to the tissue repairing induced by larval migration [33]. Of note, inflammatory responses in the liver were associated with control of parasitic infection as observed in animals resistant to *A*. *suum* infection [34]. Some cytokines released by eosinophils such as TNF (a hallmark of inflammatory response) could also be implicated, among other important physiological functions, in tissue remodelling [35]. Interestingly, some previous studies have demonstrated that TNF plays a key role in the expulsion of helminth *T*. *muris* while working synergistically with a Th2 response, especially IL-13 [36, 37].

Models of resistance and susceptibility have been studied for gastrointestinal helminths. Usually, protection against reinfection is mediated by the Th2-type response, where as susceptibility is associated with a pro-inflammatory response [37, 38]. Our data demonstrated that reinfected mice showed an increased production of IL-17A, IL-6, TNF-α, IL-4 and IL-5, suggesting a pattern of mixed Th2/Th17 responsiveness, which was previously observed in studies with helminths [8–10, 39, 40] and also allergic disorders, such as rhinitis and asthma [41]. The production of IL-10 in the reinfected group as previously demonstrated during *T*. *muris* and *S*. *mansoni* infections [42], however, might be possibly related to immunomodulation of Th1 or Th17 inflammatory responses [42–44]. Indeed, the increased levels of IL-17A in multiple exposures to *A*. *suum* might reflect the intense and chronic inflammation in the tissue remodeling, as is well demonstrated in a model of pulmonary fibrosis [45]. Among the pleiotropic mechanisms of action, this cytokine acts on neutrophil recruitment and helps in elimination of bacteria associated with the surface of the parasite and also in phagocytosis of cellular debris present in the areas of necrosis [46]. Moreover, IL-17A contributes to granulomatous inflammatory and fibrosing reactions in animals infected with *S*. *japonicum* [47] or animals continuous exposured to *Aspergillus fumigatus* [48].

It is noteworthy that reinfected animals exhibited significantly higher levels of IL-4 until the beginning stage (fourth day) of the last infection. As the increased expression of receptors for IL-4 induces the expression of IL-10 [46], the combination of these two cytokines is crucial for the control of wound damage caused by migration of *Nippostrongylus braziliensis* larvae through the organs of the host. Thus, IL-10 might control inflammation as IL-4 may mediate tissue healing by promoting the response from macrophages and eosinophils [42, 43, 46, 49]. Moreover, while previous studies demonstrated that IL-4 and IL-10 have inhibitory effects on IL-17A [30, 40], the presence of systemic production of these cytokines in animals that were repeatedly exposured to *A*. *suum* was not yet clear.

The coexistence of a mixed Th2/Th17 in reinfected mice with increased levels of IL-4 and IL-17A after each infection might also be associated to tissue injury and remodeling. Indeed, it has been shown that these cytokines have a role in development of lung fibrosis in response to chronic tissue injury [45, 50]. Morever, the robust Th2/Th17 response may reduce larval burden in multiple exposures, thus playing a dual role by acting direct on leukocytes to induce aspecific immune response against *Ascaris* and by inducing tissue thickening and fibrosis that frustrate worm migration. As observed in the histopathological analysis, the repeated exposure to the parasite could cause large areas of damage in the liver and lungs with concurrent increased host inflammatory response by eosinophis and neutrophils and hence a need for regulation of immune responses. Interestingly, the histophatological analysis showed that single-infected mice presented higher levels of hemoglobin and proteins in the BAL, even when the same animals had less damage to areas in pulmonary tissue. Possibly, during the first parasitic infection, when the protective response observed after multiple exposures are not yet effective, a higher number of *Ascaris* larvae migrates through the lungs and airwarys aggravating the destruction of capillary vessels and causing tissue damage, exudation and extravasation of proteins in the BAL.

Associated with increased tissue damage and exudation caused by larval migration, substantial changes in pulmonary physiology such as loss of pulmonary volume, airway flow and elasticity were observed as a consequence of intense parenchymal injury and aedema in single-infected mice. After multiple exposures to *Ascaris*, the persistence of physiological modulation and the chronic and repetitive lung parenchymal injury with intense eosinophilic immune responses are consistent with human larval ascariasis associated with Loeffler's syndrome/eosinophilic pneumonitis [51, 52]. Our data suggests the development of pulmonary fibrosisas a cumulative effect of larval ascariasis and eosinophil persistence in tissue, proceding to restrictive and non-reversible lung disease. Moreover, the injury in pulmonary parenchyma and tissue remodeling by fibrogenesis might lead to a progressive increase in pulmonary resistance, detected either in single or multiple infections, as a consequence of tissue scarring induced by multiple larval migrations into airways and anti-helminthic Th2/Th17 and pro-fibrogenic immune responses.

Taken together, our data indicate that, after multiple larval ascariasis, the host is able to mount a protective response against reinfection. Such a finding may explain the worldwide epidemiological distribution of *Ascaris* given the majority of infected individuals in areas of high endemicity suffer only low parasitic burden. Furthermore, this study suggests the intense systemic and pulmonary inflammatory responses that occur after repeated exposures might be fundamental to induction of protection. However, in the same scenario, the intense airway and lung inflammation that is triggered to control larval migration may be responsible for respiratory malfunction, possibly even asthma, in a host that has been multiply exposed to the parasite. This hypothesis might explain instances of seasonal eosinophilic pneumonitis and asthma among Saudi Arabs exposed to *Ascaris* sp. larvae [53]. While data from our study might explain the differences in indicators of inflammation in putatively immune (probably comprising multiply exposed individuals) versus susceptible (likely suffering the first exposure) children [54], further studies are required to provide more evidence about the biology of the interaction between *Ascaris* and the host, focusing mainly focused on elucidation of the immune mechanisms and pathways of protection that are triggered to control larval ascariasis burdens and tissue damage. This could enable the development of new strategies to prevent or treat *Ascaris* infection.
